# Long-term impact of sarcopenia on functional decline and mortality in community-dwelling older adults: a systematic review and meta-analysis

**DOI:** 10.3389/fnut.2025.1652386

**Published:** 2026-01-08

**Authors:** Yuan Zhao, Yueying Jiang, Yunyu Guo, Wanya Pan, Wenhao Tian, Leiwen Tang, Xiuqin Feng

**Affiliations:** Department of Nursing, The Second Affiliated Hospital Zhejiang University School of Medicine, Hangzhou, China

**Keywords:** sarcopenia, aging, functional decline, mortality, community-dwelling, meta-analysis

## Abstract

**Background:**

Sarcopenia is an age-related syndrome characterized by progressive loss of muscle mass and function. While it is considered a key predictor of adverse health outcomes, comprehensive evidence regarding its long-term impact on functional decline and mortality in community-dwelling older adults remains limited.

**Objective:**

To evaluate the longitudinal association between baseline sarcopenia and risks of functional decline and all-cause mortality among community-dwelling older adults, with subgroup analyses based on methods of sarcopenia assessment and domains of functional decline.

**Methods:**

We conducted a systematic review and meta-analysis following PRISMA 2020 and MOOSE guidelines. Seven databases were searched from inception to September 30^th^, 2025. We included cohort studies of older adults aged 60 years and above, with sarcopenia defined by recognized criteria, and reporting effect estimates for functional decline or mortality with follow-up for 1 year or longer. A meta-analysis based on heterogeneity was conducted using either common or random-effects models.

**Results:**

A total of 39 studies involving 76151 participants were included. Sarcopenia was significantly associated with an increased risk of all-cause mortality (29 publications, OR = 1.79, 95%CI: 1.55~2.06) and functional decline (16 publications, OR = 1.90, 95%CI: 1.55~2.32). Subgroup analyses revealed consistent associations across different muscle mass assessment methods (DXA, BIA, and CT). Notably, sarcopenia was associated with both physical (OR = 1.91, 95%CI: 1.52~2.40) and cognitive/psychological functional decline (OR = 2.03, 95%CI: 1.35~3.05). Heterogeneity was moderate to high but did not substantially alter the results in sensitivity analyses.

**Conclusion:**

This meta-analysis confirms that sarcopenia significantly predicts long-term functional decline and mortality in community-dwelling older adults, with robust associations across multiple muscle mass measurement methods and functional domains. These findings highlight the importance of standardized sarcopenia screening and early intervention to mitigate long-term functional impairment and mortality risk in aging populations.

**Clinical trial registration:**

PROSPERO (ID CRD42024595362).

## 1 Introduction

Sarcopenia is a progressive neogeriatric syndrome characterized by age-related loss of muscle mass, loss of muscle strength, and/or reduced somatic function, with a high prevalence, insidious onset, and can have widespread systemic effects (1, 2). Individuals begin to lose muscle mass from around 40 years of age, and the prevalence of sarcopenia is expected to become more common in older adults as life expectancy rises (3). By 2050, there will be 500 million sarcopenia sufferers worldwide, making it one of the main public health issues. Unfortunately, there is no global consensus on the assessment and diagnosis of sarcopenia, and the prevalence of sarcopenia varies greatly among different countries and regions. The findings of the most recent global epidemiological survey on sarcopenia indicate that the prevalence of sarcopenia in older adults aged ≥65 ranges from 5.7% ~ 33%, and it can reach as high as 50% ~ 60% in those aged≥80 (1).

Research has indicated that lifestyle and environment are the main determinants of sarcopenia (4). The pathogenesis of sarcopenia is complex (5), involving factors such as neuronal loss, mitochondrial dysfunction, hormonal fluctuations, nutritional inadequacies, or dietary modifications. Furthermore, sarcopenia interacts with chronic diseases, increasing the likelihood of adverse outcomes like falls, frailty, disability, and mortality in addition to the high expenses of care and healthcare associated with these conditions (3, 6–8). Globally, no consistent diagnostic standards for sarcopenia have been developed. Many attempts have been made to standardize cutoff thresholds and criteria for sarcopenia based on physical function, strength, and muscle mass. Given the possibility of reversibility, a deeper comprehension of sarcopenia is essential for the creation of novel disease-preventive techniques in the future.

While numerous studies have linked sarcopenia to poor health outcomes, most existing reviews have been narrative or limited in scope, often focusing on specific populations, short-term outcomes, or single health domains. A comprehensive quantitative synthesis of the long-term predictive value of sarcopenia for functional decline and mortality in community-dwelling older adults is still lacking. Therefore, this systematic review and meta-analysis aims to quantitatively evaluate the long-term impact of baseline sarcopenia on functional decline and all-cause mortality among community-dwelling older adults. By integrating evidence from cohort studies with follow-up durations of one year or more, and by conducting in-depth subgroup analyses, this study seeks to provide robust, stratified evidence to inform early screening, preventive strategies, and person-centered care in nursing and public health practice.

## 2 Methods

This systematic review and meta-analysis were performed according to the Preferred Reporting Items for Systematic Review and Meta-Analysis (PRISMA) guidelines 2020. Please see the Supplementary material for a detailed checklist. We have registered on PROSPERO (identifier ID CRD42024595362). This study adhered to the Meta-analysis of Observational Studies in Epidemiology (MOOSE) report guideline (9).

### 2.1 Search strategy

A systematic search was undertaken up to September 30^th^, 2025 through the following databases: PubMed, EBSCOhost (APA PsycInfo and CINAHL with full text), the Excerpta Medica dataBASE (EMBASE), Web of Science (WOS), Scopus, and the Cochrane Library. The initial search strategy was performed using keywords, MeSH terms, and free text words such as “Sarcopenia,” “Aged/Frail elderly/Older,” “Community-dwelling,” “Mortality/Functional decline/Frailty/Disability.” Moreover, keywords and subject headings were exhaustively combined using Boolean operators without time restriction and in humans only. We performed manual reference checking of the included studies and relevant review articles to identify any additional eligible records. The complete search strategies for each database are shown in Table 1.

**Table 1:** Search strategies and results.

Database	Search strategies
PubMed	#1 "Sarcopenia"[Mesh] OR sarcopeni*[tiab] OR "muscle wasting"[tiab] OR "muscle loss"[tiab] OR "low muscle mass"[tiab] OR "skeletal muscle index"[tiab] #2 "Aged"[Mesh] OR "Aged, 80 and over"[Mesh] OR "Frail Elderly"[Mesh] OR elder*[tiab] OR older[tiab] OR senior*[tiab] OR geriatric[tiab] OR "oldest old"[tiab] #3 "Independent Living"[Mesh] OR "community dwelling"[tiab] OR "community-dwelling"[tiab] OR "living at home"[tiab] OR "outpatient"[tiab] OR "primary care"[tiab] #4 #2 AND #3 #5 "Activities of Daily Living"[Mesh] OR "Disability Evaluation"[Mesh] OR "Frailty"[Mesh] OR "Mortality"[Mesh] OR "functional decline"[tiab] OR "ADL disability"[tiab] OR "IADL disability"[tiab] OR frail*[tiab] OR mortality[tiab] OR death[tiab] OR survival[tiab] #6 "Prognosis"[Mesh] OR "Cohort Studies"[Mesh] OR "Longitudinal Studies"[Mesh] OR "Follow-Up Studies"[Mesh] OR prognostic[tiab] OR predict*[tiab] OR cohort[tiab] OR longitudinal[tiab] OR prospective[tiab] OR retrospective[tiab] OR "follow-up"[tiab] #7 #1 AND #4 AND #5 AND #6 791
EBSCO host (APA PsycInfo, CINAHL with full text)	(Sarcopenia OR "muscle wasting" OR "muscle loss" OR "low muscle mass" OR "skeletal muscle index") AND ("Aged" OR "Aged, 80 and over" OR "Frail Elderly" OR elderly OR older OR senior OR geriatric OR "oldest old") AND ("Independent Living" OR "community dwelling" OR "community-dwelling" OR "living at home" OR "outpatient" OR "primary care") AND ("Activities of Daily Living" OR "Disability Evaluation" OR Frailty OR Mortality OR "functional decline" OR "ADL disability" OR "IADL disability" OR frail OR death OR survival) AND ("Prognosis" OR "Cohort Studies" OR "Longitudinal Studies" OR "Follow-Up Studies" OR prognostic OR predict OR cohort OR longitudinal OR prospective OR retrospective OR "follow-up") 226
Web of science	1: TS=(Sarcopenia OR "muscle wasting" OR "muscle loss" OR "low muscle mass" OR "skeletal muscle index") 41449 2: TS=("Aged" OR "Aged, 80 and over" OR "Frail Elderly" OR elderly OR older OR senior OR geriatric OR "oldest old") 3214115 3: TS=("Independent Living" OR "community dwelling" OR "community-dwelling" OR "living at home" OR "outpatient" OR "primary care") 409937 4: #3 AND #2 110517 5: TS=("Activities of Daily Living" OR "Disability Evaluation" OR Frailty OR Mortality OR "functional decline" OR "ADL disability" OR "IADL disability" OR frail OR death OR survival) 4046512 6: TS=("Prognosis" OR "Cohort Studies" OR "Longitudinal Studies" OR "Follow-Up Studies" OR prognostic OR predict OR cohort OR longitudinal OR prospective OR retrospective OR "follow-up") 7139968 7: #1 AND #4 AND #5 AND #6 696
Embase	#1 'sarcopenia':ti,ab OR 'muscle wasting':ti,ab OR 'muscle loss':ti,ab OR 'muscle weakness'/exp OR 'muscle weakness' OR 'low muscle mass':ti,ab OR 'skeletal muscle index':ti,ab 570292 #2 'aged'/exp OR 'frail elderly'/exp OR 'elderly':ti,ab OR 'older':ti,ab OR 'senior':ti,ab OR 'geriatric':ti,ab OR 'oldest old':ti,ab 5,129,178 #3 'independent living'/exp OR 'community dwelling person'/exp OR 'community-dwelling':ti,ab OR 'community dwelling':ti,ab OR 'living at home':ti,ab OR 'outpatient':ti,ab OR 'primary care':ti,ab OR 'primary health care'/exp 717,506 #4 #2 AND #3 202,693 #5 'activities of daily living':ti,ab OR 'daily life activity'/exp OR 'disability evaluation':ti,ab OR 'disability assessment'/exp OR 'frailty'/exp OR 'mortality'/exp OR 'functional decline':ti,ab OR 'adl disability':ti,ab OR 'iadl disability':ti,ab OR 'frail':ti,ab OR 'death':ti,ab OR 'survival'/exp OR 'disability'/exp 4,362,917 #6 'longitudinal study'/exp OR 'cohort':ti,ab OR 'prospective':ti,ab OR 'retrospective':ti,ab OR 'cohort study':ti,ab OR 'follow-up':ti,ab OR 'longitudinal':ti,ab OR 'prognostic':ti,ab OR 'prognosis'/exp OR 'predict':ti,ab 6,962,705 #7 #1 AND #4 AND #5 AND #6 998
Cochrane library	#1 (Sarcopenia OR "muscle wasting" OR "muscle loss" OR "low muscle mass" OR "skeletal muscle index"):ti,ab,kw 4142 #2 ("Aged" OR "Aged, 80 and over" OR "Frail Elderly" OR elderly OR older OR senior OR geriatric OR "oldest old"):ti,ab,kw AND ("Independent Living" OR "community dwelling" OR "community-dwelling" OR "living at home" OR "outpatient" OR "primary care"):ti,ab,kw 35590 #3 ("Activities of Daily Living" OR "Disability Evaluation" OR Frailty OR Mortality OR "functional decline" OR "ADL disability" OR "IADL disability" OR frail OR death OR survival):ti,ab,kw 296495 #4 ("Prognosis" OR "Cohort Studies" OR "Longitudinal Studies" OR "Follow-Up Studies" OR prognostic OR predict OR cohort OR longitudinal OR prospective OR retrospective OR "follow-up"):ti,ab,kw 669271 #5 #1 AND #2 AND #3 AND #4 58
Scopus	TITLE-ABS-KEY (Sarcopenia OR "muscle wasting" OR "muscle loss" OR "low muscle mass" OR "skeletal muscle index") AND TITLE-ABS-KEY ("Aged" OR "Aged, 80 and over" OR "Frail Elderly" OR elderly OR older OR senior OR geriatric OR "oldest old") AND TITLE-ABS-KEY ("Independent Living" OR "community dwelling" OR "community-dwelling" OR "living at home" OR "outpatient" OR "primary care") AND TITLE-ABS-KEY ("Activities of Daily Living" OR "Disability Evaluation" OR Frailty OR Mortality OR "functional decline" OR "ADL disability" OR "IADL disability" OR frail OR death OR survival) AND TITLE-ABS-KEY ("Prognosis" OR "Cohort Studies" OR "Longitudinal Studies" OR "Follow-Up Studies" OR prognostic OR predict OR cohort OR longitudinal OR prospective OR retrospective OR "follow-up") 705

### 2.2 Inclusion and exclusion criteria

The study will be included if it meets all of the following PI(/E)COS criteria: ① Population (P): Community-dwelling older adults (mean age≥60 years) at baseline. Studies focusing exclusively on institutionalized (e.g., nursing home residents) or hospitalized patients will be excluded. ②Exposure (E): Sarcopenia, defined at baseline by either: 1. A recognized international operational definition (e.g., the European Working Group for Sarcopenia 2014/2019, the European Working Group on Sarcopenia in Older People 1/2, the International Working Group on Sarcopenia, the Foundation for the National Institute of Health). 2. Objective measures of low muscle mass (e.g., via DXA, BIA, CT, MRI) combined with low muscle strength (e.g., handgrip strength) and/or low physical performance (e.g., SPPB, gait speed). ③ Comparator (C): Community-dwelling older adults without sarcopenia. ④ Outcomes (O): At least one of the following longitudinal outcomes must be reported: 1. Functional Decline (New onset of physical, psychological, cognitive, and social functional decline). 2. Mortality. ⑤ Study Design (S): Cohort studies with a minimum follow-up period of 1 year.

Study exclusion Criteria: Studies will be excluded for any of the following reasons: Studies with a cross-sectional design. Studies that do not report original data (e.g., reviews, editorials, protocols). Studies that do not provide a clear definition of sarcopenia or sufficient data to ascertain the exposure. Studies that do not report adjusted effect estimates, like Hazard Ratio (HR), Odds Ratio (OR), Relative Risk (RR) or provide sufficient raw data to calculate these estimates for the association between sarcopenia and the outcomes of interest.

If a single study reported multiple sarcopenia definitions from different guidelines (like AWGS and EWGSOP), all applicable definitions were included as independent estimates in the meta-analysis. In cases where multiple definitions were available from the same guideline organization (such as AWGS 2014 and AWGS 2019), we prioritized the most recent version (AWGS 2019) for the primary analysis to maintain consistency with current diagnostic standards. Study-specific cutoffs were only considered if they aligned with a recognized international definition or were validated against one.

### 2.3 Study selection and data extraction

**Figure 1:**
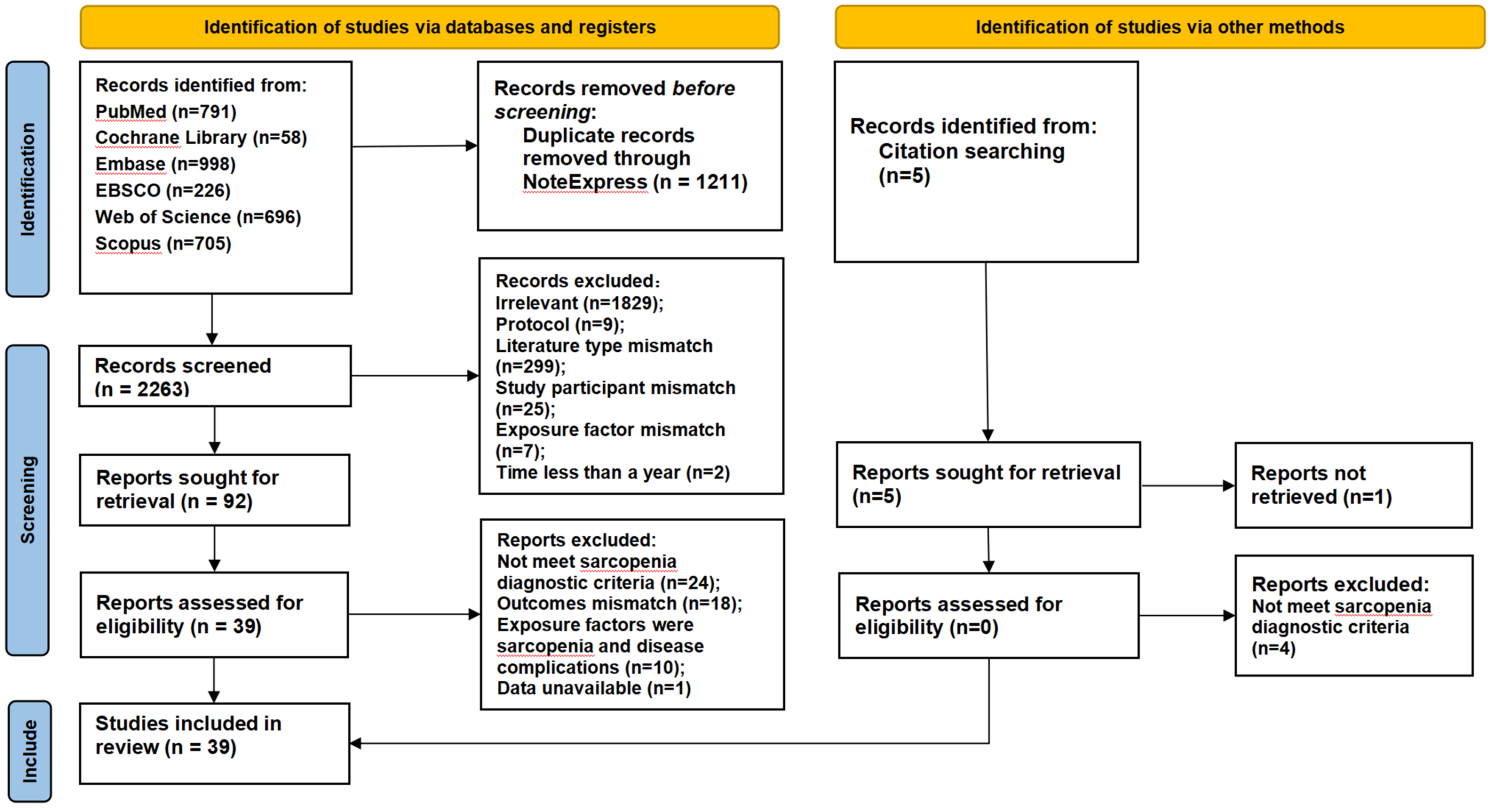
PRISMA flow diagram.

Two reviewers independently reviewed each title and abstract for eligibility based on the inclusion and exclusion criteria. If the study was considered suitable, the full text was retrieved. Full-text articles were assessed for final inclusion. Data extraction was the next step if, after reading the entire text, the article was still deemed appropriate for the study. For clarification, the lead author was contacted if any data were unclear or missing. To gather data from articles that qualified, a pre-established data collection form was utilized. The form collected the following information: first author and publication year, study design, population’s age range and sex ratio, sample size, length of follow-up, definition criteria for sarcopenia, assessment instruments of sarcopenia and outcomes (if available), and effect size for the association between sarcopenia and outcomes. The study selection process is summarized in Figure 1. Detailed search strategies are provided in Table 1, characteristics of the included studies are shown in Table 2, and the quality assessment results are presented in Table 3. A pre-specified set of study-level covariates was extracted to explore potential sources of clinical heterogeneity. These characteristics for all included studies are summarized in Supplementary Table S1.

**Table 2:** Characteristics of the studies.

Study	Location	Study design	Age range (mean ± sd, sex %)	Sample size	Follow-up	Exposure factor characteristics	Outcome characteristics	Effect size
Shim et al. (2025) (29)	Korea	Retrospective cohort study	75.6 ± 7.5, 51% male	594	15 years	Sarcopenia definition: KWGS Muscle mass: DXA Grip strength: Dynamometer Physical performance: SPPB	① Mortality; ② Mobility limitation	① HR = 1.512, 95% CI: 1.054 ~ 2.169; ② OR = 3.461, 95% CI: 1.965 ~ 6.121；
Frisoli Junior et al. (2025) (45)	Brazil	Prospective cohort study	78 ± 7.3, 58.5% female	439	18 months	Sarcopenia definition: SDOC criteria, EWGSOP 2 criteria Muscle mass: DXA Grip strength: Dynamometer Physical performance: 4.5 m gait speed	Mortality	SDOC criteria: HR = 5.444, 95% CI:1.943 ~ 14.060; EWGSOP 2 criteria: HR = 2.789, 95% CI: 0.318 ~ 24.412;
Yao et al. (2024) (20)	USA	Prospective cohort study	73.8 ± 6.2, 51.9% female	416	2.9 years	Sarcopenia definition: CT cutoffs validated in healthy US populations Muscle mass: CT Grip strength: Dynamometer Physical performance: gait speed (6-m course)	Mortality	L3-SMA: HR = 1.25, 95% CI:1.11 ~ 1.54; T12-SMA: HR = 1.32, 95% CI:1.11 ~ 1.56; T12-SMI: HR = 1.19, 95% CI:1 ~ 1.43;
Nakano et al. (2024) (53)	Japan	Prospective cohort study	76 (69–81), 35% female	251	5.2 years	Sarcopenia definition: AWGS 2014 Muscle mass: DXA Grip strength: Dynamometer Physical performance: gait speed	Mortality	HR = 1.66, 95% CI: 0.72 ~ 3.83
Ramoo et al. (2024) (35)	Malaysia	Prospective cohort study	≥60, 62.4% female	2,404	83 months	Sarcopenia definition: AWGS 2019 Muscle mass: BIA Grip strength: Dynamometer Physical performance: 4-m gait speed	Mortality	HR = 2.14, 95% CI: 1.59 ~ 2.87
Yamaguchi et al. (2023) (16)	Japan	Prospective cohort study	73.7 ± 6.1, 53% female	13,569	5 years	Sarcopenia definition: EWGSOP 2, AWGS 2019 Muscle mass: BIA Grip strength: Dynamometer Physical performance: 2.4-m distance gait speed	Mortality	HR = 1.66, 95% CI: 1.11 ~ 2.48
Oh et al. (2023) (21)	Korea	Prospective cohort study	75.9 ± 3.9, 47.2% male	1959	2 years	Sarcopenia definition: AWGS 2019 Appendicular skeletal mass: DXA Grip strength: Dynamometer Physical performance: UGS, 5-STS test, SPPB	IADL disabilities	OR = 2.77, 95% CI: 1.21 ~ 6.33
Peng et al. (2023) (27)	China	Prospective cohort study	75.8 ± 4.8, 45.7% men	394	2 years	Sarcopenia definition: AWGS 2019, EWGSOP2 Muscle mass: BIA Grip strength: Dynamometer Physical performance: 8 feet distance	Cognitive function	AWGS 2019: OR = 1.56，95%CI: 0.77 ~ 2.17 EWGSOP2: OR = 0.2, 95%CI: 0.013 ~ 2.99
Liang et al. (2023) (31)	China	Prospective cohort study	73.4 ± 5.4, 52.8% male	731	11 years	Sarcopenia definition: AWGS 2019, AWGS 2014 Appendicular skeletal mass: DXA Grip strength: Dynamometer Physical performance: 6-meter walk test	Mortality	AWGS 2019: HR = 1.62, 95% CI: 1.04 ~ 2.54; AWGS 2014: HR = 1.61, 95% CI: 0.99 ~ 2.63;
Ulugerger Avci et al. (2023) (48)	Turkey	Retrospective cohort study	76.1 ± 6.4, 68.6% female	175	5 years	Appendicular skeletal mass: BIA Grip strength: Dynamometer Physical performance: 6-meter gait speed	Mortality	HR = 2.26, 95% CI: 1.15 ~ 4.43
Zhang et al. (2023) (50)	China	Prospective cohort study	65 (62 ~ 69), 46.5% female	2,689	4 years	Sarcopenia definition: AWGS 2019 Appendicular skeletal muscle mass: equation Grip strength: Dynamometer Physical performance: 2.5-m walking course, chair stand test	Cognitive function	OR = 1.56, 95%CI: 1.2 ~ 2
Li et al. (2022) (30)	China	Prospective cohort study	≥65, 54.6% male	641	12 years	Sarcopenia definition: EWGSOP 1 height-adjusted skeletal muscle index (hSMI): DXA Handgrip strength (HS): Dynamometer Physical performance: gait speed (GS), timed up-and-go test (TUG), timed chair stand (TCS), weight-adjusted leg press (WaLP)	Mortality	low hSMI and low GS: HRs = 4.33, 95% CI: 2.76 ~ 6.78; low hSMI and high TUG: HRs = 4.11, 95% CI: 2.60 ~ 6.48; low hSMI and high TCS: HRs = 2.97, 95% CI: 1.92 ~ 4.59; low hSMI and low WaLP: HRs = 3.19, 95% CI: 2.13 ~ 4.79; low hSMI and low HS: HRs = 4.08, 95% CI: 2.70 ~ 6.17
Pereira et al. (2022) (38)	Brazil	Prospective cohort study	70.0 ± 6.3, 39.4% male	132	10 years	Sarcopenia definition: EWGSOP 1 criteria, EWGSOP 2 criteria Muscle mass: DXA	Mortality	EWGSOP1: HR = 2.01, 95%CI: 1.03 ~ 3.92; EWGSOP2: HR = 2.07, 95%CI: 1.05 ~ 4.06;
Lenchik et al. (2021) (18)	North Carolina	Retrospective cohort study	63.7 ± 2.8, 59.9% male	11,361	6 years	Sarcopenia definition: a crowd-validated automated calculation method Skeletal muscle area: CT	Mortality	Men: HR = 0.83, 95%CI: 0.77 ~ 0.90; Women: HR = 0.97, 95%CI: 0.85 ~ 1.09
Chiba et al. (2021) (23)	Japan	Retrospective cohort study	75.5 ± 4.0, 55.7% female	2,149	5 years	Sarcopenia definition: AWGS 2019 Appendicular skeletal mass: BIA Grip strength: Dynamometer Physical performance: 2.4-m distance gait speed	Disability	HR = 2.31, 95%CI: 1.48 ~ 3.62
Thompson et al. (2021) (26)	Australia	Prospective cohort study	74.1 ± 6.1, 55.5% female	716	10 years	Sarcopenia definition: EWGSOP 1 criteria Muscle mass: DXA Grip strength: Dynamometer	Mortality	HR = 1.65, 95%CI: 0.85 ~ 3.20
Cawthon et al. (2021) (33)	Portland	Prospective cohort study	84.2 ± 4.1, 100% male	1,425	① 2.2 years; ② 2.2 years; ③ 3.3 years;	Sarcopenia definition: EWGSOP 2 Lean mass: D3-Creatine Dilution Method	① ADL disability ② IADL disability ③ Mortality	D3-Creatine Dilution: ① RR = 1.9, 95% CI: 1.2 ~ 3.1; ② RR = 1.5, 95% CI: 1.3 ~ 1.9; ③ RR = 1.8, 95% CI: 1.5 ~ 2.2;
Takahashi et al. (2021) (43)	Japan	Prospective cohort study	71.3 ± 6.3, 58.6% female	396	40.5 months	Sarcopenia definition: AWGS 2019 Skeletal muscle mass index: DXA Grip strength: Dynamometer	Mortality	HR = 6.12, 95%CI: 1.52 ~ 24.7
Ishii et al. (2020) (24)	Japan	Prospective cohort study	73.5 ± 5.5, 47.6% male	9,229	2 years	Sarcopenia definition: AWGS 2014 Muscle mass: BIA	Disability	HR = 4.03, 95% CI: 2.85 ~ 5.70
Costanzo et al. (2020) (28)	Italy	Prospective cohort study	77 ± 5.5, 53.6% female	535	3 years	Sarcopenia definition: agreement between EWGSOP 1 criteria and EWGSOP 2 criteria Muscle mass: BIA Grip strength: Dynamometer Physical performance: timed chair stand	① Disability ② Mortality	① RR = 2.43, 95%CI: 0.38 ~ 8.53; ②HR = 1.96, 95%CI: 0.63 ~ 6.15;
Bachettini et al. (2020) (40)	Brazil	Prospective cohort study	≥60, 62.6% female	1,291	2.6 years	Sarcopenia definition: EWGSOP 1& 2 criteria Muscle mass: calf circumference (CC) Grip strength: Dynamometer Physical performance: 4-meter distance	Mortality	EWGSOP 1 criteria: HR = 1.18, 95%CI: 0.53 ~ 2.65; EWGSOP 2 criteria: HR = 1.36, 95%CI: 0.52 ~ 3.57;
De Santana et al. (2019) (15)	Brazil	Prospective cohort study	73.0 ± 5.2, 61.5% female	839	4 years	Sarcopenia definition: EWGSOP 2 criteria Appendicular Lean Mass: DXA	Mortality	OR = 4.21, 95% CI: 1.80 ~ 9.87
Björkman et al. (2019) (19)	Finland	Prospective cohort study	83.4 ± 4.6, 66.6% female	428	4 years	Sarcopenia definition: EWGSOP 2 cr iteria Muscle mass: BIA Grip strength: Dynamometer Physical performance: gait speed, SPPB	Mortality	HR = 1.82, 95% CI: 1.09 ~ 3.03
Uemura et al. (2019) (36)	Japan	Prospective cohort study	71.9 ± 5.4, 48.9% male	4,452	30 months	Sarcopenia definition: AWGS 2014 Muscle mass: BIA Grip strength: Dynamometer Physical performance: gait speed	Disability	HR = 2.74, 95% CI: 1.58 ~ 4.77
Sim et al. (2019) (42)	Australia	Prospective cohort study	79.9 ± 2.6,	903	① 5 years ② 9.5 years	Sarcopenia definition: FNIH, EWGSOP 1, adapted FNIH, adapted EWGSOP 1 Muscle mass: DXA Grip strength: Dynamometer Physical performance: TUG	Mortality	① 5 years: EWGSOP 1: aHR = 1.88. 95%CI: 1.24 ~ 2.85; adapted EWGSOP 1: aHR = 2.52, 95%CI: 1.55 ~ 4.09; FNIH: aHR = 1.08, 95% CI: 0.56 ~ 2.08; adapted FNIH: aHR = 1.03, 95%CI: 0.56 ~ 1.89; ② 9.5 years: EWGSOP 1: aHR = 1.39. 95%CI: 1.06 ~ 1.81; adapted EWGSOP 1: aHR = 1.94, 95%CI: 1.40 ~ 2.69; FNIH: aHR = 1.35, 95% CI: 0.93 ~ 1.95; adapted FNIH: aHR = 1.09, 95%CI: 0.77 ~ 1.56
Sobestiansky et al. (2019) (46)	Sweden	Prospective cohort study	86.6 ± 1.0, 100% male	287	3 years	Sarcopenia definition: FNIH, EWGSOP 1, EWGSOP 2 Muscle mass: DXA	Mortality	EWGSOP 1: HR = 1.95, 95%CI: 1.12 ~ 3.40; EWGSOP 2: HR = 1.70, 95%CI: 0.94 ~ 3.05; FNIH: HR = 1.65, 95%CI: 0.73 ~ 3.72;
Wang et al. (2019) (47)	China	Prospective cohort study	93.5 ± 3.2, 32.2% male	738	4 years	Sarcopenia definition: validated in a Chinese population Skeletal muscle mass: an equation	① Mortality ② ADL disability	① Women: HR = 1.54, 95%CI: 1.10 ~ 2.16; Man: HR = 0.82, 95%CI: 0.45 ~ 1.47; ② Women: HR = 1.54, 95%CI: 1.01 ~ 1.76; Man: HR = 1.73, 95%CI: 1.13 ~ 2.63;
Locquet et al. (2019) (51)	Belgium	Prospective cohort study	73.5 ± 6.2, 60.5% female	534	3 years	Sarcopenia definition: EWGSOP 1 Muscle mass: DXA Grip strength: Dynamometer Physical performance: SPPB	① Disability ② Mortality	①OR = 1.70, 95%CI: 0.87 ~ 3.35 (Cruz-Jentoft et al. Definition); OR = 1.18, 95%CI: 0.64 ~ 2.18 (Fielding et al. Definition); OR = 1.55, 95%CI: 0.69 ~ 3.45 (Morley et al. Definition); OR = 2.98, 95%CI: 0.98 ~ 7.07 (Chen et al. Definition); OR = 1.74, 95%CI: 0.72 ~ 4.22 (Studenski et al. Definition); ② HR = 2.93, 95%CI: 1.17 ~ 7.35 (Cruz-Jentoft et al. Definition); HR = 2.94, 95%CI: 1.19 ~ 7.25 (Fielding et al. Definition); HR = 2.79, 95%CI: 1.11 ~ 5.07 (Morley et al. Definition); HR = 4.43, 95%CI: 1.64 ~ 11.96 (Chen et al. Definition); HR = 2.35, 95%CI: 0.63 ~ 8.78 (Studenski et al. Definition);
Chen et al. (2019) (52)	China	Prospective cohort study	67.5 ± 5.7, 44% men	691	1 year	Sarcopenia definition: AWGS 2014 Muscle mass: DXA Grip strength: Dynamometer Physical performance: gait speed	Psychological function	OR = 3.57, 95%CI: 1.59 ~ 8.04
Jang et al. (2018) (22)	Korea	Prospective cohort study	76, 55.2% female	1,343	22 months	Sarcopenia definition: KNHANES Muscle mass: BIA Grip strength: Dynamometer Physical performance: 4-m distance gait speed	① Mortality ② ADL Disability ③ IADL Disability	① Men: HR = 5.18, 95%CI: 3.03 ~ 8.85 (ASM/ht^2^); HR = 1.77, 95%CI: 1.02 ~ 3.08 (ASM/wt); HR = 3.33, 95%CI: 1.89 ~ 5.88 (ASM/BMI); Women: HR = 2.16, 95%CI: 1.28 ~ 3.65 (ASM/ht^2^); HR = 1.48, 95%CI: 0.83 ~ 2.67 (ASM/wt); HR = 1.63, 95%CI: 0.93 ~ 2.85 (ASM/BMI); ②OR = 1.30, 95%CI: 0.81 ~ 2.07 (ASM/ht^2^); OR = 1.42, 95%CI: 0.90 ~ 2.26 (ASM/wt); OR = 1.65, 95%CI: 1.05 ~ 2.59 (ASM/BMI); ③ OR = 2.15, 95%CI: 1.30 ~ 3.57 (ASM/ht^2^); OR = 2.50, 95%CI: 1.51 ~ 4.15 (ASM/wt); OR = 2.08, 95%CI: 1.26 ~ 3.45 (ASM/BMI);
Ting-Ching Tang et al. (2018) (25)	China	Prospective cohort study	73.4 ± 5.4, 52.9% males	728	32.9 months	Sarcopenia definition: FNIH lean mass: DXA Grip strength: Dynamometer Physical performance: 6-meter gait speed	Mortality	HR = 3.8, 95%CI: 1.26 ~ 11.45 (Weak + Low ALM/BMI); HR = 3.85, 95%CI: 1.25 ~ 11.85 (Weak_BMI_ + Low ALM/BMI);
Uemura et al. (2018) (39)	Japan	Prospective cohort study	71.8 ± 5.4, 49.8% men	3,958	15 months	Sarcopenia definition: AWGS 2014 Muscle mass: BIA Grip strength: Dynamometer Physical performance: 2.4-m walking path	Physiological function	OR = 2.02, 95%CI: 1.06 ~ 3.85
Balogun et al. (2017) (34)	Australia	Prospective cohort study	63 ± 7.5, 50% female	1,041	10 years	Sarcopenia definition: cutoffs of low muscle mass that has been validated by US populations Appendicular lean mass: DXA Grip strength: Dynamometer Physical performance: Dynamometer	Mortality	RR = 1.17, 95%CI: 0.85 ~ 1.66 (ALM/ht^2^); RR = 1.54, 95%CI: 1.14 ~ 2.08 (ALM/BMI); RR = 1.25, 95%CI: 0.91 ~ 1.72 (ALM/wt);
Pérez-Zepeda et al. (2017) (39)	Mexico	Prospective cohort study	85.2 ± 6.4, 72% female	172	1 year	Sarcopenia definition: EWGSOP 1 Muscle mass: BIA	Mortality	HR = 2.23, 95% CI: 1.15 ~ 4.34
Yuki et al. (2017) (41)	Japan	Prospective cohort study	71.4 ± 0.5, 50.7% male	720	11 years	Sarcopenia definition: AWGS 2014 Muscle mass: DXA Grip strength: Dynamometer Physical performance: 10-meter distance	Mortality	Men: HR = 1.90, 95% CI:1.04 ~ 3.46; Women: HR = 0.77, 95% CI:0.29 ~ 2.22;
Brown et al. (2016) (37)	USA	Prospective cohort study	70.1 ± 0.14, 43.5% male	4,425	14.4 years	Sarcopenia definition: EWGSOP 1 Muscle mass: BIA Physical performance: 4-meter gait speed	Mortality	HR = 1.29, 95%CI: 1.13 ~ 1.47
Hirani et al. (2015) (44)	Australia	Prospective cohort study	77 (70 ~ 97), 100% men	1705	①5 years ②7 years	Sarcopenia definition: FNIH Muscle mass: DXA Grip strength: Dynamometer Physical performance: 4-meter gait speed	① ADL disability ② Mortality	① OR = 2.77 95%CI: 1.30 ~ 5.87 (low ALM alone); OR = 3.78, 95%CI: 1.23 ~ 11.64 (low ALM with weakness); OR = 4.53, 95%CI: 0.90 ~ 22.72 (sarcopenia with weakness and poor gait speed); ② HR = 1.65 95%CI: 1.30 ~ 2.09 (low ALM alone); HR = 1.50, 95%CI: 1.08 ~ 2.08 (low ALM with weakness); HR = 1.69, 95%CI: 1.17 ~ 2.44 (sarcopenia with weakness and poor gait speed);
Tanimoto et al. (2013) (17)	Japan	Prospective cohort study	73.2 ± 6.1, 34.2% male	716	2 years	Muscle mass: BIA Grip strength: Dynamometer Physical performance: gait speed	ADL disability	Men: OR = 45.1, 95%CI: 6.5 ~ 313.9 Women: OR = 10.4, 95%CI: 1.8 ~ 59.8
Amigues et al. (2013) (32)	France	Retrospective cohort study	79.9 ± 3.5, 100% female	975	4 years	Skeletal muscle mass: DXA	IADL disability	aHR = 1.29, 95% CI: 0.98 ~ 1.72

KWGS, Korean Working Group on Sarcopenia Guideline; SDOC, Sarcopenia Definition and Outcomes Consortium; AWGS 2019, Asian Working Group for Sarcopenia 2019; DXA, dual-energy X-ray absorptiometry; BIA, bioelectrical impedance analysis; SPPB, Short Physical Performance Battery; ADL, activities of daily living; IADL, instrumental ADL; FNIH, the United States Foundation for the National Institutes of Health; ALM/ht^2^, appendicular lean mass (ALM)/height^2^ (kg/m^2^); ALM/BMI, low ALM/body mass index (kg/kg/m^2^); ALM/wt, ALM/weight*100 (kg/kg); TUG: time up and go.

**Table 3:** Newcastle–Ottawa scale.

Publication	Study design	selection				comparability		Outcome/Exposure			Overall
		a1	a2	a3	a4	b1	b2	c1	c2	c3	
Shim et al. (2025) (29)	Retrospective cohort study	*	*	*	*	*	*		*	*	8*
Frisoli Junior et al. (2025) (45)	Prospective cohort study	*	*	*	*	*	*	*	*	*	9*
Yao et al. (2024) (20)	Prospective cohort study	*	*	*	*	*		*	*	*	8*
Nakano et al. (2024) (53)	Nakano et al. (2024) (53)		*	*	*	*	*	*	*	*	8*
Ramoo et al. (2024) (35)	Prospective cohort study	*	*	*	*	*	*	*	*	*	9*
Yamaguchi et al. (2023) (16)	Prospective cohort study	*	*	*	*	*	*	*	*	*	9*
Oh et al. (2023) (21)	Prospective cohort study	*	*	*	*	*	*	*	*	*	9*
Peng et al. (2023) (27)	Prospective cohort study	*	*	*	*	*	*	*	*	*	9*
Liang et al. (2023) (31)	Prospective cohort study	*	*	*	*	*	*	*	*	*	9*
Ulugerger Avci et al. (2023) (48)	Retrospective cohort study	*	*	*	*	*		*	*		7*
Zhang et al. (2023) (50)	Prospective cohort study	*	*	*	*	*	*	*	*	*	9*
Li et al. (2022) (30)	Prospective cohort study	*	*	*		*	*	*	*	*	8*
Pereira et al. (2022) (38)	Prospective cohort study	*	*	*	*	*	*	*	*	*	9*
Lenchik et al. (2021) (18)	Retrospective cohort study	*	*	*	*	*	*	*	*	*	9*
Chiba et al. (2021) (23)	Retrospective cohort study	*	*	*	*	*	*	*	*	*	9*
Thompson et al. (2021) (26)	Prospective cohort study	*	*	*	*	*	*	*	*		8*
Cawthon et al. (2021) (33)	Prospective cohort study	*	*	*	*	*	*	*	*	*	9*
Takahashi et al. (2021) (43)	Prospective cohort study	*	*	*	*	*	*	*	*		8*
Ishii et al. (2020) (24)	Prospective cohort study	*	*	*	*	*	*	*	*	*	9*
Costanzo et al. (2020) (28)	Prospective cohort study	*	*	*	*	*	*	*	*	*	9*
Bachettini et al. (2020) (40)	Prospective cohort study	*	*	*	*	*	*	*	*	*	9*
De Santana et al. (2019) (15)	Prospective cohort study	*	*	*	*	*	*	*	*	*	9*
Björkman et al. (2019) (19)	Prospective cohort study	*	*	*	*	*	*	*	*		8*
Uemura et al. (2019) (36)	Prospective cohort study	*	*	*	*	*	*	*	*	*	9*
Sim et al. (2019) (42)	Prospective cohort study	*	*	*	*			*	*	*	7*
Sobestiansky et al. (2019) (46)	Prospective cohort study	*	*	*	*	*	*	*	*	*	9*
Wang et al. (2019) (47)	Prospective cohort study	*	*	*	*	*	*	*	*	*	9*
Locquet et al. (2019) (51)	Prospective cohort study	*	*	*	*	*	*	*	*	*	9*
Chen et al. (2019) (52)	Prospective cohort study	*	*	*	*	*	*		*	*	8*
Jang et al. (2018) (22)	Prospective cohort study	*	*	*	*	*	*	*	*	*	9*
Tang et al. (2018) (25)	Prospective cohort study	*	*	*	*	*	*	*	*	*	9*
Uemura et al. (2018) (49)	Prospective cohort study	*	*	*	*	*	*		*	*	8*
Balogun et al. (2017) (34)	Prospective cohort study	*	*	*	*	*	*	*	*	*	9*
Pérez-Zepeda et al. (2017) (39)	Prospective cohort study		*	*	*	*	*	*	*	*	8*
Yuki et al. (2017) (41)	Prospective cohort study	*	*	*	*	*	*	*	*	*	9*
Brown et al. (2016) (37)	Prospective cohort study	*	*	*	*	*	*	*	*	*	9*
Hirani et al. (2015) (44)	Prospective cohort study		*	*	*	*	*	*	*	*	8*
Tanimoto et al. (2013) (17)	Prospective cohort study		*	*	*	*			*	*	6*
Amigues et al. (2013) (32)	Retrospective cohort study	*	*	*	*	*	*		*	*	8*

Guidelines for the evaluation of the Newcastle Ottawa Scale are detailed in the “Supplementary material.” One publication may include several health outcomes, we defined that a publication that included one or more self-reported outcomes was not scored “*” for the item c1 in the NOS score； In the same cohort, with less than 6-month of follow-up for any of the outcomes, item c2 was evaluated as no “*.” In the same cohort, with dropout rate>20% and no description provided of those lost for any of the outcomes, item c3 was evaluated as no “*.”

### 2.4 Methodological quality assessment

The Newcastle–Ottawa Scale, a 9-star instrument for assessing bias, was used to further evaluate the quality of all included research. 0 ~ 3 stars, 4 ~ 6 stars, and 7 stars and above are classified as low-quality, medium-quality, and high-quality literature, respectively (10). To lower the risk of bias, two researchers separately extracted the data. The third researcher, who also decided on the study inclusion criteria, assessed all search results and review procedures after the data extraction process was finished. In the outcome measurement, we determined that a follow-up duration of at least 1 year was sufficient for the occurrence of an event of interest. We stipulated that a dropout rate of ≤20% would not significantly affect the results, making the cohort follow-up adequate.

### 2.5 Statistical analysis

Forest plots were generated using R version 4.4.1 to show the effect size of the chosen studies in our meta-analysis and to visualize the results. The effect size of every included study was utilized in the forest plot to pool an overall effect summary using a suitable effect model. The heterogeneity between studies was tested using I^2^ statistics. Based on the I^2^ value, a suitable effect summary model was selected after the heterogeneity analysis was finished. Significant heterogeneity was considered if the I^2^ value was ≥50% (or *p* value <0.10), in which case the random-effects model was used. If the I^2^ value was <50%, we considered that the heterogeneity was acceptable, and the fixed-effects model was applied (11, 12). The summary effect size was expressed as Odds Ratios (ORs) with their 95% Confidence Intervals (95%CIs), and the pooled results value indicated the impact of sarcopenia on outcomes including functional decline and mortality. Higher risk was generally indicated by a pooled OR values greater than 1. A review of the relevant literature in this field reveals significant heterogeneity among different studies. To explore potential sources of clinical heterogeneity beyond measurement tools and functional domains, we pre-specified the following study-level covariates for subgroup analysis or meta-regression where feasible: geographic region (Asia, Europe, Americas, etc.), mean/median age, sex, follow-up duration, sarcopenia diagnostic criteria, muscle mass assessment tool, outcome domain, and whether the analysis adjusted for key confounders such as age, sex, comorbidities, medication, smoking, or drinking. However, due to the limited number of studies for some outcomes and covariates, meta-regression was not performed for all subgroups. Instead, we relied on stratified subgroup analyses and sensitivity analyses to assess the robustness of the findings. A sensitivity analysis of the included studies was carried out by eliminating one study at a time to assess the robostness of the pooled effect size estimate. Publication bias was evaluated using Egger’s test and visual inspection of funnel plot asymmetry when there were more than 10 included studies for an outcome (13, 14).

## 3 Results

### 3.1 Search process

Details of the search results are presented in Table 1. There were 3,474 records found in the first search. 2,263 titles and abstracts were screened after duplicates (*n* = 1,211) were eliminated. Of these, 92 were chosen to undergo a full-text evaluation to determine eligibility (Figure 1 PRISMA flow diagram). An additional 53 studies were eliminated based on the inclusion and exclusion criteria during full-text review. As a result, 39 studies were ultimately included to this systematic review for narrative synthesis or meta-analysis.

### 3.2 Characteristics of studies-narrative synthesis

A total of 39 studies (15–53) were included in this systematic review, corresponding to 76,151 older adults. Overall, 34 were prospective cohort studies and 5 were retrospective cohort studies. Studies were conducted between 2013 and 2025. The follow-up period ranged from 1 to 15 years. Most of the studies (20/39) were carried out in Asia (16, 17, 21–25, 27, 29–31, 35, 36, 41, 43, 47, 49, 50, 52, 53), while 8 studies originated from Europe (18, 19, 28, 32, 33, 46, 48, 51), 4 from Brazil (15, 38, 40, 45), 4 from Australia (26, 34, 42, 44), and 3 from North America (20, 37, 39). Dual-energy X-ray absorptiometry (DXA) was the instrument most often used to assess muscle mass (19 studies, 48.7%) (15, 21, 25, 26, 29–32, 34, 38, 41–46, 51–53), followed by Bioelectrical impedance analysis (BIA) (14 studies, 35.9%) (16, 17, 19, 22–24, 27, 28, 35–37, 39, 48, 49), CT scan (2 studies) (18, 20) and anthropometric equation (2 studies) (47, 50), calf circumference (CC) (1 study) (40), and D3-Creatine Dilution Method (1 study) (33). The most commonly used diagnostic criteria for sarcopenia are the AWGS 2014/2019 and EWGSOP 1/2. For specific descriptive information on the final included studies, please refer to Table 2.

### 3.3 Quality assessment

With 24 studies receiving a rating of nine stars, 12 receiving eight stars, two receiving seven stars, and one receiving six stars, the included studies were generally of high quality. The primary reasons for failing to achieve a perfect score in study quality assessment include failure to adjust for confounding factors, outcome measurement lacking blinding or relying solely on patient-reported methods, and insufficient population representativeness. Please see Table 3 for details of the specific quality evaluation results.

### 3.4 Meta-analysis

#### 3.4.1 Sarcopenia and mortality

**Figure 2:**
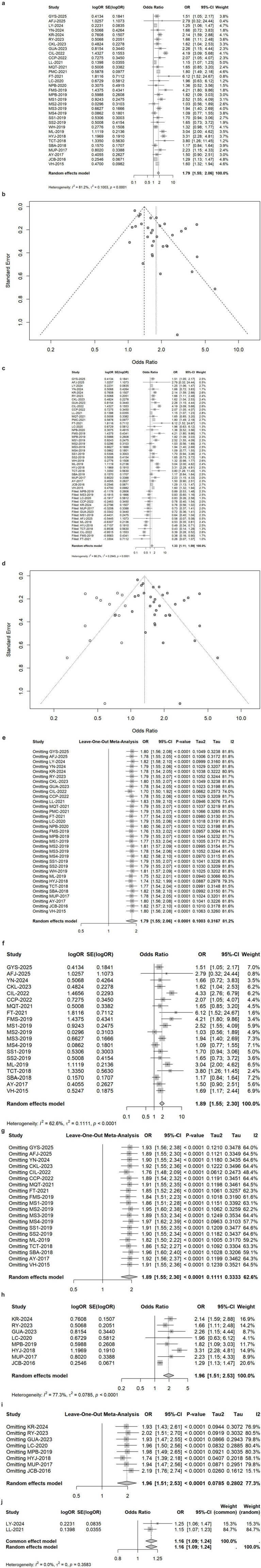
**(a)** Forest plot of the predictive efficacy of sarcopenia on mortality. **(b)** Funnel plot of publication bias risk in predicting mortality with sarcopenia. **(c)** Forest plot of the predictive efficacy of sarcopenia on mortality (after trim-and-fill). **(d)** Funnel plot of publication bias risk in predicting mortality with sarcopenia (after trim-and-fill). **(e)** Sensitivity analysis plot of sarcopenia in predicting mortality efficacy. **(f)** Forest plot of the predictive efficacy of sarcopenia on mortality (using DXA to measure muscle mass). **(g)** Sensitivity analysis plot of sarcopenia in predicting mortality efficacy (using DXA to measure muscle mass). **(h)** Forest plot of the predictive efficacy of sarcopenia on mortality (using BIA to measure muscle mass). **(i)** Sensitivity analysis plot of sarcopenia in predicting mortality efficacy (using BIA to measure muscle mass). **(j)** Forest plot of the predictive efficacy of sarcopenia on mortality (using CT to measure muscle mass).

From 29 publications (15, 16, 18–20, 22, 25, 26, 28–31, 33–35, 37–48, 51, 53) (26 prospective publications, 3 retrospective publications, *n* = 48,939) that provided data on the association between sarcopenia and mortality. The final pooled result of 33 independent studies showed an increased mortality risk for community-dwelling older adults with sarcopenia, compared with those without sarcopenia (OR = 1.79,95%CI: 1.55 ~ 2.06). The heterogeneity between studies was significant (I^2^ = 81.2%, *p*_heterogeneity_<0.0001) (Figure 2a). The Egger’s test (bias estimate = 2.04, *p* = 0.0002) and funnel plot (Figure 2b) provided possible evidence for publication bias. Fifteen studies were added to the trim-and-fill analysis, and the significance of the summary relative risk did not change substantially (OR = 1.33, 95%CI: 1.11 ~ 1.59, I^2^ = 86.2%, *p*_heterogeneity_ = 0.0016) (Figure 2c). Furthermore, publication bias was not detected after trim-and-fill according to Egger’s test (bias estimate = 0.39, *p* = 0.51) (Figure 2d). To assess the excessive influence of individual studies on the pooled effect size, we conducted a leave-one-out analysis. The result showed that after sequentially excluding each study from the analysis, the pooled ORs ranged from 1.7 (95%CI: 1.5 ~ 1.92) to 1.83 (95%CI: 1.59 ~ 2.10). This showed no substantial difference in either magnitude or direction compared to our primary analysis (OR = 1.79, 95%CI: 1.55 ~ 2.06). Furthermore, the heterogeneity statistics remained stable, with the I^2^ statistic fluctuating between 73.4 and 81.8% (I^2^ = 81.2% in the primary analysis), indicating that our primary findings are robust to the influence of any single study (Figure 2e).

Due to significant heterogeneity, we conducted a subgroup analysis. Following data extraction from the literature, we discovered that one of the main causes of significant variation was the measurement of muscle mass. All studies were divided into six subgroups based on the various methods used to measure muscle mass: DXA, BIA, CT, equation-based, calf circumference-based, and D3-Creatine Dilution Method. When two or more publications were included in each subgroup, meta-analyses were conducted. 16 publications (15, 25, 26, 29–31, 34, 38, 41–46, 51, 53) employing DXA demonstrated that, compared to community-dwelling older adults without baseline sarcopenia, those with sarcopenia exhibited a 1.89-fold increased risk of mortality (OR = 1.89, 95%CI: 1.55 ~ 2.30), with moderate heterogeneity (I^2^ = 62.6%, *p*_heterogeneity_<0.0001) (Figure 2f). Sensitivity analyses yielded similarly robust results (OR [95% CI] range: 1.76 [95%CI: 1.48 ~ 2.09] to 1.97 [95%CI: 1.62 ~ 2.39], I^2^ = 48.4% ~ 64.6%). Eight publications used BIA (16, 19, 22, 28, 35, 37, 39, 48), the final pooled results were OR = 1.96, 95%CI: 1.51 ~ 2.53 (I^2^ = 77.3%, *p*_heterogeneity_<0.0001) (Figure 2h). Through sensitivity analysis, we identified one study as the primary source of heterogeneity. After excluding this study, heterogeneity decreased to 15.1%. The final pooled results still showed statistically significant, with OR = 2.19, 95% CI: 1.76 ~ 2.74. Two publications measured muscle mass using CT scans (18, 20). The pooled results showed an OR = 1.16 (95% CI: 1.09 ~ 1.24), with no evidence of heterogeneity (I^2^ = 0, *p*_heterogeneity_ = 0.36) (Figure 2j). The remaining three methods each had only one included publication, making meta-analysis inapplicable. A comprehensive summary of all sensitivity analyses, including leave-one-out ranges, trim-and-fill adjustments, and heterogeneity statistics, is provided in Supplementary Table S2.

### 3.5 Sarcopenia and functional decline

**Figure 3:**
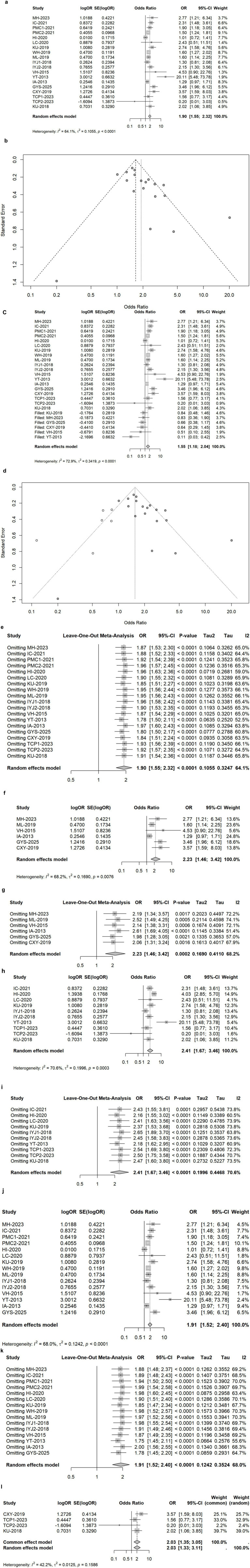
**(a)** Forest plot of the predictive efficacy of sarcopenia on functional decline. **(b)** Funnel plot of publication bias risk in predicting functional decline with sarcopenia. **(c)** Forest plot of the predictive efficacy of sarcopenia on functional decline (after trim-and-fill). **d)** Funnel plot of publication bias risk in predicting functional decline with sarcopenia (after trim-and-fill). **(e)** Sensitivity analysis plot of sarcopenia in predicting functional decline efficacy. **(f)** Forest plot of the predictive efficacy of sarcopenia on fucntional decline (using DXA to measure muscle mass). **(g)** Sensitivity analysis plot of sarcopenia in predicting functional decline efficacy (using DXA to measure muscle mass). **(h)** Forest plot of the predictive efficacy of sarcopenia on fucntional decline (using BIA to measure muscle mass). **(i)** Sensitivity analysis plot of sarcopenia in predicting functional decline efficacy (using BIA to measure muscle mass). **(j)** Forest plot of the predictive efficacy of sarcopenia on physical function. **(k)** Sensitivity analysis plot of sarcopenia in predicting physical function efficacy. **(l)** Forest plot of the predictive efficacy of sarcopenia on psychological/cognitive function.

16 publications (17, 21–24, 27–29, 32, 33, 36, 44, 47, 49, 51, 52) (14 prospective publications, 2 retrospective publications) were included in the meta-analysis, with 33,492 participants in total. With a pooled estimate of OR = 1.9 (95%CI: 1.55 ~ 2.32, I^2^ = 64.1%, *p _heterogeneity_*<0.0001), sarcopenia was associated with a higher risk of functional decline among community-dwelling older adults (Figure 3a). Egger’s test (bias estimate = 1.62, *p* = 0.02) and the funnel plot (Figure 3b) provided possible evidence for publication bias. Six studies were added through the trim-and-fill method, and the summary result still remained significant (OR = 1.55, 95%CI: 1.18 ~ 2.04, I^2^ = 72.9%, *p*_heterogeneity_<0.0001) (Figure 3c). The new Egger’s test showed statistically insignificant (bias estimate: 0.11, *p* = 0.73), with the new symmetric funnel plot (Figure 3d). Sensitivity analyses showed robust results (Figure 3e), with insignificant fluctuations in heterogeneity and significance after the exclusion of any single study (OR = 1.78, 95%CI: 1.5 ~ 2.11 to OR = 1.97, 95%CI: 1.6 ~ 2.43, I^2^ range from 52.5 to 66.1%).

We conducted subgroup analyses based on different muscle mass measurement tools. The combined results for the DXA (21, 29, 32, 44, 51, 52) and BIA (17, 22–24, 27, 28, 36, 49) groups showed OR_DXA_ = 2.23, 95% CI: 1.46 ~ 3.42; OR_BIA_ = 2.41, 95% CI: 1.67 ~ 3.46, respectively (Figures 3f,h). This indicates higher sensitivity when using BIA for disability risk assessment, though it may also overestimate disability risk. Both subgroup analysis results exhibited substantial heterogeneity (I^2^ = 68.2%, I^2^ = 70.6%, *p _heterogeneity_* < 0.01) and sensitivity analysis results remained robust (Figures 3g,i). This could be the result of notable differences in the methods used for assessment across several functional domains, such as physical, cognitive, and psychological functions. The D3-Creatine Dilution Method and equation-based methods were used in the other two publications to measure muscle mass, rendering meta-analysis unnecessary.

Similarly, we conducted subgroup analyses based on the multidimensionality of functional decline. The combined odds ratio (OR) for physical functional decline (17, 21–24, 28, 29, 32, 33, 36, 44, 47, 51) in community-dwelling older individuals with baseline sarcopenia was 1.91 (95% CI: 1.52 ~ 2.40), with significant heterogeneity (I^2^ = 68%, *p _heterogeneity_* < 0.0001), according to the results (Figure 3j). Sensitivity analysis yielded robust results (OR [95% CI]: 1.75 [1.45 ~ 2.11] to 2.00 [1.56 ~ 2.55]; I^2^: 55.6 to 70.3%) (Figure 3k). The pooled effect size for cognitive or psychological decline in older adults with baseline sarcopenia was OR = 2.03, 95% CI: 1.35 ~ 3.05. The results of the heterogeneity analysis showed no significant difference (I^2^ = 42.2%, *p _heterogeneity_* = 0.16) (Figure 3l).

## 4 Discussion

This systematic review and meta-analysis, comprising 39 longitudinal studies with 76,151 community-dwelling older adults, provides robust quantitative evidence that baseline sarcopenia significantly increases the long-term risks of both functional decline (OR = 1.90) and all-cause mortality (OR = 1.79). Notably, these associations remained consistent across multiple subgroup and sensitivity analyses, reinforcing the prognostic value of sarcopenia in aging populations.

Our findings align with prior systematic reviews (54–60) that reported sarcopenia as a risk factor for adverse outcomes. However, this study extends existing evidence in several critical ways. First, while earlier meta-analyses often focused on a certain population like cancer patients, a single outcome such as cardiovascular disease or cognitive impairment, our work simultaneously quantifies the impact of sarcopenia on multidimensional functional decline—including physical, cognitive, and psychological domains—and mortality within the same analytical framework. Second, by restricting inclusion to studies with a minimum one-year follow-up, we specifically addressed the long-term impact of sarcopenia, a dimension less emphasized in previous reviews. Third, a key innovative aspect of our analysis lies in the in-depth exploration of heterogeneity through subgroup analyses based on muscle mass measurement methods. We found that while the association between sarcopenia and mortality was significant across all tools, the magnitude varied: BIA showed the highest pooled OR (1.96), followed by DXA (1.89), and CT (1.16). This suggests that the choice of assessment tool may influence risk stratification, possibly due to differences in sensitivity, specificity, or the underlying physiological parameters measured. Similarly, for functional decline, both DXA and BIA subgroups yielded strong associations (OR = 2.23 and 2.41, respectively), indicating that sarcopenia is a robust predictor irrespective of the measurement technique, though BIA might offer higher sensitivity in community settings.

Sarcopenia is significantly associated with functional decline and mortality, underscoring the necessity of implementing early comprehensive management strategies. Building upon our findings and former relevant studies, we propose the following structured, actionable recommendations for integrating sarcopenia screening into clinical practice, stratified by resource settings and individual risk: (1) Systematic screening in primary care: We recommend implementing systematic screening for adults aged ≥70 years (or ≥60 for high-risk individuals, sunch as those with multimorbidity, recent falls, or unexplained weight loss) during routine primary care visits (61). Initial screening should prioritize functional assessments: handgrip strength (using portable dynamometers) or usual gait speed (over 4 ~ 6 meters) every 1 ~ 2 years. In low-resource settings or where equipment is limited, calf circumference (< 34 cm in men, < 33 cm in women) offers a highly feasible and cost-effective initial tool. (2) Referral and confirmatory testing: Individuals identified as high-risk during initial screening (like handgrip strength below sex-specific thresholds or gait speed < 0.8 m/s) should be referred for confirmatory assessment. Bioelectrical impedance analysis (BIA) provides a practical balance of accuracy, accessibility, and cost for community settings. Where available and feasible, dual-energy X-ray absorptiometry (DXA) remains the reference standard for quantifying muscle mass. Standardized diagnostic thresholds, aligned with international criteria (e.g., EWGSOP2, AWGS 2019), should be applied consistently to ensure comparability across settings, acknowledging that heterogeneity in measurement methods necessitates context-appropriate calibration. (3) Integration with chronic disease management: Given the synergistic relationship between sarcopenia and chronic conditions such as diabetes, cardiovascular disease, or cancer, screening and management should be embedded within existing care models for these diseases. For instance, incorporating sarcopenia assessment into annual diabetes reviews or cardiac rehabilitation programs can identify at-risk individuals earlier and facilitate integrated interventions targeting shared pathways of functional decline. (4) Considerations for policy and implementation: While our meta-analysis reports a pooled odds ratio of 1.79 for mortality and 1.90 for functional decline, translating these relative risks into absolute risk reductions and population impact requires individual participant data from cohort studies, which was not available for this analysis. Future research should aim to provide such absolute risk estimates to better inform public health planning and calculate numbers needed to screen. Furthermore, the trim-and-fill adjusted effect sizes (OR = 1.33 for mortality; OR = 1.55 for functional decline), while still statistically significant, suggest a more conservative true effect than the initial estimates. This uncertainty should be explicitly considered when modeling the long-term benefits, cost-effectiveness, and potential resource implications of widespread screening programs. In addition to the structured screening approach outlined above, multimodal interventions are essential and should include: resistance-based exercise programs to improve muscle mass and strength (62), nutritionally appropriate protein and vitamin D supplementation (63–65), as well as cognitive and psychosocial support integrated into physical rehabilitation (66–68). Nurses and community health workers play a pivotal role in conducting initial assessments, delivering education, and coordinating care across settings.

### 4.1 Limitations

Several limitations should be considered when interpreting our findings. First, significant heterogeneity was observed across the meta-analyses (I^2^ > 50%), which may stem from variations in sarcopenia definitions, differences in outcome measurement tools, and population diversity. Although we conducted subgroup analyses to explore these sources, residual heterogeneity remains. The observed heterogeneity in muscle mass assessment methods underscores the need for standardized, context-appropriate diagnostic protocols in clinical practice. Second, although the association between sarcopenia and mortality (OR = 1.33, 95%CI: 1.11 ~ 1.59) or functional decline (OR = 1.55, 95%CI: 1.18 ~ 2.04) remained statistically significant after trim-and-fill adjustment, the magnitude of the pooled effect decreased substantially from the initial estimate (OR_mortality_ = 1.79; OR_functional decline_ = 1.9). This suggests that the original meta-analysis may have been influenced by small-study effects or publication bias, leading to an overestimation of the true effect. While the direction and significance of the association remain unchanged, the more conservative effect size should be considered when interpreting the clinical and public health implications. This uncertainty should be considered when modeling the long-term benefits and cost-effectiveness of widespread screening programs. It is also important to note that funnel plot asymmetry may arise from sources other than publication bias, such as heterogeneity in study design, outcome measurement, or selective reporting. Third, most included studies were observational, limiting causal inference. Finally, the limited number of studies using emerging methods such as the D3-Creatine Dilution Method prevented their inclusion in subgroup meta-analyses, highlighting an area for future research. Furthermore, future studies could also incorporate validated questionnaires to assess quality of life, psychosocial well-being, and daily affairs, which may provide deeper insights into the lived experience of older adults with sarcopenia.

## 5 Conclusion

In conclusion, this study establishes sarcopenia as a critical determinant of long-term functional decline and mortality in community-dwelling older adults. By integrating evidence across sarcopenia measurement methods and functional domains, we provide a comprehensive foundation for early screening, personalized intervention, and policy development aimed at preserving independence and quality of life in an aging global population.

## Data Availability

The original contributions presented in the study are included in the article/Supplementary material, further inquiries can be directed to the corresponding author/s.
