# Supplementary material for: Long-term impact of sarcopenia on functional decline and mortality in community-dwelling older adults: a systematic review and meta-analysis
Source: Front Nutr. 2026 Jan 8;12:1652386. doi: 10.3389/fnut.2025.1652386 (PMC12823505; doi:10.3389/fnut.2025.1652386)
Supplement: Supplementary file 3 [file Table_3.DOCX]

**Table S1 Study-level Covariates**

| Study | Region | Study design | Participants characteristics (mean age, % female) | Follow-up duration (Years) | Sarcopenia Assessment | | Outcome Measures | | Adjusted Covariates |
| --- | --- | --- | --- | --- | --- | --- | --- | --- | --- |
|  |  |  |  |  | Diagnosis criteria | Muscle mass tool | Functional decline | Mortality |  |
| Ga Yang Shim et al, 2025 | Korea (Asia) | Retrospective cohort study | 75.6±7.5, 49% female | 15 | KWGS | DXA | Mobility limitation | Mortality | Age, BMI, KIADL, GDS, MMSE, regular exercise, smoking, drinking, CCI |
| Alberto Frisoli Junior et al, 2025 | Brazil (South America) | Prospective cohort study | 78±7.3, 58.5 % female | 1.5 | SDOC, EWGSOP 2 | DXA | Not reported | Mortality | Age, falls, hearing loss |
| Lawrence Yao et al, 2024 | USA (North America) | Prospective cohort study | 73.8±6.2, 51.9% female | 2.9 | CT cutoffs validated in US populations | CT | Not reported | Mortality | Age |
| Yuta Nakano  et al, 2024 | Japan (Asia) | Prospective cohort study | 76 (69-81), 35% female | 5.2 | AWGS 2014 | DXA | Not reported | Mortality | Age, sex, BMI, diabetes, cardiovascular disease, chronic kidney disease stage, serum albumin |
| Karthikeyanathan Ramoo et al, 2024 | Malaysia (Asia) | Prospective cohort study | ≥60, 62.4% female | 6.9 | AWGS 2019 | BIA | Not reported | Mortality | Age, sex, depression, pain, visual impairment, hearing loss |
| Ryo Yamaguchi et al, 2023 | Japan (Asia) | Prospective cohort study | 73.7±6.1, 53% female | 5 | EWGSOP 2, AWGS 2019 | BIA | Not reported | Mortality | Age, BMI, MMSE, smoking, drinking, comorbidities, polypharmacy |
| Min Hyung Oh et al, 2023 | Korea (Asia) | Prospective cohort study | 75.9±3.9, 52.8% female | 2 | AWGS 2019 | DXA | IADL disabilities | Not reported | Age, sex, clinical centre, smoking, physical activities, nutritional status, BMI, comorbidities |
| Tao-Chun Peng et al, 2023 | China (Asia) | Prospective cohort study | 75.8±4.8, 54.3% female | 2 | AWGS 2019, EWGSOP 2 | BIA | Cognitive function | Not reported | Age, sex, education, physical activities, smoking, drinking, diabetes, hypertension, hyperlipidemia, depression, follow-up duration |
| C-K Liang et al 2023 | China (Asia) | Prospective cohort study | 73.4±5.4, 47.2% female | 11 | AWGS 2019, AWGS 2014 | DXA | Not reported | Mortality | Age, sex, education, drinking, disease burden, testosterone levels |
| Gulru Ulugerger Avci et al, 2023 | Turkey  (Europe) | Retrospective cohort study | 76.1±6.4, 68.6% female | 5 | EWGSOP 2 | BIA | Not reported | Mortality | Age |
| Jiajia Zhang et al, 2023 | China (Asia) | Prospective cohort study | 65 (62~69), 46.5% female | 4 | AWGS 2019 | Equation | Cognitive function | Not reported | Age, sex, lifestyle, clinical factors |
| Chia-Ing Li et al, 2022 | China (Asia) | Prospective cohort study | ≥65, 45.4% female | 12 | EWGSOP 1 | DXA | Not reported | Mortality | Age, sex, education, lifestyle, comorbidities |
| Cristina Camargo Pereira et al, 2022 | Brazil (South America) | Prospective cohort study | 70.0±6.3, 60.6% female | 10 | EWGSOP 2 | DXA | Not reported | Mortality | Age, sex, BMI, smoking, physical activities, diabetes |
| Leon Lenchik et al, 2021 | North Carolina (Europe) | Retrospective cohort study | 63.7±2.8, 40.1% female | 6 | Crowd-validated automated calculation method | CT | Not reported | Mortality | Age, race, height, weight, smoking, diabetes, COPD, cardiovascular disease, cancer |
| Ippei Chiba et al, 2021 | Japan (Asia) | Retrospective cohort study | 75.5±4.0, 55.7% female | 5 | AWGS 2019 | BIA | Disability | Not reported | Age, sex, BMI, education, MMSE, comorbidities, depression, polypharmacy |
| Mark Q Thompson et al, 2021 | Australia | Prospective cohort study | 74.1±6.1, 55.5 % female | 10 | EWGSOP 1 | DXA | Not reported | Mortality | Age, sex, education, income, comorbidities |
| Peggy M Cawthon et al, 2021 | Portland (Europe) | Prospective cohort study | 84.2±4.1, 0% female | ①2.2  ②2.2  ③3.3 | EWGSOP2 | D3-Creatine dilution method | ①ADL disability  ②IADL disability | Mortality | Age, race, clinical center, comorbidities, BMI, MMSE, lifestyle |
| Fuyuko Takahashi et al, 2021 | Japan (Asia) | Prospective cohort study | 71.3±6.3, 58.6% female | 3.4 | AWGS 2019 | DXA | Not reported | Mortality | Age, sex, diabetes duration, heart disease, cancer, smoking, exercise, drinking, medication, hypertension, BMI, triglycerides, creatinine, HbA1c |
| Hideaki Ishii et al, 2020 | Japan (Asia) | Prospective cohort study | 73.5 ± 5.5, 52.4% female | 2 | AWGS 2014 | BIA | Disability | Not reported | Age, sex, MMSE, depression, pain, IADL, comorbidities, polypharmacy |
| Luisa Costanzo et al, 2020 | Italy (Europe) | Prospective cohort study | 77±5.5, 53.6% female | 3 | EWGSOP 2 | BIA | Disability | Mortality | Age, sex, BMI, marriage, education, comorbidities |
| Nathalia Perleberg Bachettini et al, 2020 | Brazil (South America) | Prospective cohort study | ≥60, 62.6% female | 2.6 | EWGSOP 2 | Calf circumference | Not reported | Mortality | Age, sex, BMI, marriage, occupation status, comorbidities, smoking, physical activities, depression |
| Felipe M de Santana et al, 2019 | Brazil (South America) | Prospective cohort study | 73.0±5.2, 61.5% female | 4 | EWGSOP 2 | DXA | Not reported | Mortality | Age, sex, BMI, physical activity, falls, drinking, diabetes, cardiovascular events, serum phosphorus, calcium, albumin, vitamin D |
| Mikko P Björkman et al 2019 | Finland (Europe) | Prospective cohort study | 83.4±4.6, 66.6% female | 4 | EWGSOP 2 | BIA | Not reported | Mortality | Age, sex, CCI |
| Kazuki Uemura et al, 2019 | Japan (Asia) | Prospective cohort study | 71.9±5.4, 51.1% female | 2.5 | AWGS 2014 | BIA | Disability | Not reported | Age, sex, BMI, education, medication, comorbidities, MMSE, GDS |
| Marc Sim et al, 2019 | Australia | Prospective cohort study | 79.9±2.6 | ①5  ②9.5 | adapted FNIH, adapted EWGSOP 1 | DXA | Not reported | Mortality | Age |
| Sigvard Sobestiansky et al, 2019 | Sweden (Europe) | Prospective cohort study | 86.6±1.0, 0% female | 3 | FNIH, EWGSOP 2 | DXA | Not reported | Mortality | Age, CCI, education, smoking, MMSE |
| Hui Wang et al, 2019 | China (Asia) | Prospective cohort study | 93.5±3.2, 67.8% female | 4 | Validated in Chinese populations | Equation | ADL disability | Mortality | Age, sex, smoking, drinking, MMSE |
| Médéa Locquet et al, 2019 | Belgium (Europe) | Prospective cohort study | 73.5±6.2, 60.5% female | 3 | EWGSOP 1 | DXA | Disability | Mortality | Age, sex, BMI, CCI, medication, nutritional status, MMSE |
| Xiaoyu Chen et al, 2019 | China (Asia) | Prospective cohort study | 67.5±5.7, 56% female | 1 | AWGS 2014 | DXA | Psychological function | Not reported | Age, sex, BMI, education, marriage, income, sleep, comorbidities, medication, physical activities |
| Il-Young Jang et al, 2018 | Korea (Asia) | Prospective cohort study | 76, 55.2% female | 1.8 | KNHANES | BIA | ①ADL disability  ②IADL disability | Mortality | Age, sex, CCI |
| Ting-Ching Tang et al, 2018 | China (Asia) | Prospective cohort study | 73.4±5.4, 47.1% female | 2.7 | FNIH | DXA | Not reported | Mortality | Age, sex, CCI, MMSE, nutritional status |
| Kazuki Uemura et al, 2018 | Japan (Asia) | Prospective cohort study | 71.8±5.4, 50.2% female | 1.25 | AWGS 2014 | BIA | Physiological function | Not reported | Age, sex, education, comorbidities, medication, IADL limitations, physical activities, MMSE, depression, social participation |
| S Balogun et al, 2017 | Australia | Prospective cohort study | 63±7.5, 50% female | 10 | Low muscle mass cutoffs validated by North American populations | DXA | Not reported | Mortality | Age, sex, BMI |
| Mario Ulises Pérez-Zepeda et al, 2017 | Mexico (North America) | Prospective cohort study | 85.2±6.4, 72% female | 1 | EWGSOP 1 | BIA | Not reported | Mortality | Age, sex, CCI |
| Atsumu Yuki et al, 2017 | Japan (Asia) | Prospective cohort study | 71.4±0.5, 49.3% female | 11 | AWGS 2014 | DXA | Not reported | Mortality | Age, sex, CCI, leisure activities, total calorie intake, drinking, smoking |
| Justin C Brown et al, 2016 | USA (North America) | Prospective cohort study | 70.1±0.14, 56.5% female | 14.4 | EWGSOP 1 | BIA | Not reported | Mortality | Age, sex, BMI, race, education, waist circumference, smoking status, comorbidities, hospitalization history, self-rated health, healthy eating index, biomarkers, weekly walking volume |
| Vasant Hirani et al, 2015 | Australia | Prospective cohort study | 77 (70~97), 0% female | ①5  ②7 | FNIH | DXA | ADL disability | Mortality | Demographics, lifestyle, comorbidities, health conditions, blood biomarkers |
| Yoshimi Tanimoto et al, 2013 | Japan (Asia) | Prospective cohort study | 73.2±6.1, 65.8% female | 2 | EWGSOP 1 | BIA | ADL disability | Not reported | Age, BMI, CCI, education, smoking, physical activities, nutritional status |
| Isabelle Amigues et al, 2013 | France (Europe) | Retrospective cohort study | 79.9±3.5, 100% female | 4 | Low muscle mass cutoffs validated by North American populations | DXA | IADL disability | Not reported | Age, walking speed, stand-up test, balance test, comorbidities, lifestyle |

Note: KWGS: Korean Working Group on Sarcopenia Guideline; SDOC: Sarcopenia Definition and Outcomes Consortium; AWGS 2019: Asian Working Group for Sarcopenia 2019; DXA: dual-energy X-ray absorptiometry; BIA: bioelectrical impedance analysis; Mini-Mental State Examination (MMSE); Charlson Comorbidity Index (CCI); Geriatric Depression Scale (GDS); Korean Instrumental Activities of Daily Living (KIADL); body mass index (BMI); Fourth Korean National Health and Nutritional Examination Surveys (KNHANES);

**Table S2 Summary of Sensitivity Analyses and Heterogeneity Assessments**

| Analysis/Subgroup | Primary Pooled OR (95%CI) | I^2^ (p heterogeneity) | Egger’s Test (p value) | Trim-and-Fill Adjust OR (95%CI) | Leave-One-Out OR (95%CI) | Leave-One-Out I^2^ Range | Influential Studies Identified |
| --- | --- | --- | --- | --- | --- | --- | --- |
| Mortality (Overall) | 1.79 (1.55~2.06) | 81.2% (<0.0001) | 0.0002 | 1.33 (1.11~1.59) | 1.70~1.83 | 73.4%~81.8% | None |
| Mortality-DXA | 1.89 (1.55~2.30) | 62.6% (<0.0001) | - | - | 1.76~1.97 | 48.4%~64.6% | None |
| Mortality-BIA | 1.96 (1.51~2.53) | 77.3% (<0.0001) | - | - | - | - | Yes (exclusion reduced I2 to 15.1%; OR=2.19, 1,76~2.74) |
| Mortality-CT | 1.16 (1.09~1.24) | 0% (0.36) | - | - | - | - | None |
| Functional Decline (Overall) | 1.90 (1.55~2.32) | 64.1% (<0.0001) | 0.02 | 1.55 (1.18~2.04) | 1.78~1.97 | 52.5%~66.1% | None |
| Functional Decline-DXA | 2.23 (1.46~3.42) | 68.2% (<0.01) | - | - | - | - | None |
| Functional Decline-BIA | 2.41 (1.67~3.46) | 70.6% (<0.01) | - | - | - | - | None |
| Physical Function Decline | 1.91 (1.52~2.40) | 68% (<0.0001) | - | - | 1.75~2.00 | 55.6%~70.3% | None |
| Cognitive/Psychological Decline | 2.03 (1.35~3.05) | 42.2% (0.16) | - | - | - | - | None |
